# Modeling Lung Derecruitment in VILI Due to Fluid-Occlusion: The Role of Emergent Behavior

**DOI:** 10.3389/fphys.2020.542744

**Published:** 2020-10-30

**Authors:** Vitor Mori, Bradford J. Smith, Bela Suki, Jason H. T. Bates

**Affiliations:** ^1^Department of Medicine, Vermont Lung Center, Larner College of Medicine, The University of Vermont, Burlington, VT, United States; ^2^Department of Telecommunications and Control Engineering, University of São Paulo, São Paulo, Brazil; ^3^Department of Bioengineering, College of Engineering, Design & Computing, University of Colorado Denver, Aurora, CO, United States; ^4^Department of Biomedical Engineering, Boston University, Boston, MA, United States

**Keywords:** acute lung injury, analytical model, alveolar flooding, surface tension, lung elastance

## Abstract

Ventilator-induced lung injury (VILI) is driven by the processes of volutrauma and atelectrauma, which can act synergistically to compromise the blood-gas barrier. We have postulated that this synergy arises through a rich-get-richer mechanism whereby atelectrauma causes holes to form in the blood-gas barrier while concomitant volutrauma causes susceptible holes to progressively enlarge as VILI worsens. We previously developed an analytical model based on this idea that accurately predicts the progressive increases in lung elastance seen immediately following a recruitment maneuver as VILI progresses over the course of hours. In the present study we extend this model to account for the rate of change of elastance, due to closure of lung units, in the minutes following a recruitment maneuver. We found that the distribution of unit closing velocities throughout the lung can be described by a power law with an exponent of −2 that matches previously published power laws associated with the dynamics of lung recruitment. Our model thus reveals lung collapse as an example of emergent complex behavior and links the dynamics of altered function in the injured lung to structural damage in a way that explains the mechanisms of injury progression arising from the ongoing stresses and strains applied by mechanical ventilation.

## Introduction

Mechanical ventilation plays a major role in critical care, providing life support to patients in respiratory failure. However, because of the stresses and strains it imposes on the tissues of the lung, mechanical ventilation can also cause ventilator-induced lung injury (VILI) (Dos Santos and Slutsky, 2000; Gattinoni et al., 2003; Slutsky and Ranieri, 2013). The two principle injury mechanisms of VILI are over-distension of the tissues that gives rise to volutrauma, and cyclic recruitment and derecruitment of lung units that gives rise to atelectrauma (Parker et al., 1993; Dreyfuss and Saumon, 1998). Volutrauma and atelectrauma are both involved in compromising the blood-bas barrier of the lung, which allows plasma-derived fluid and proteins to leak into the airspaces. This disrupts surfactant function, which then decreases lung compliance and thus increases the tissue stresses associated with mechanical ventilation, making the leak worse. At the same time, the lung becomes progressively more derecruited, which places the remaining open lung regions at greater risk of over-distension (Gattinoni and Pesenti, 2006; Gattinoni et al., 2016). Patients with pre-existing lung injury, particularly acute respiratory system distress syndrome (ARDS), are highly susceptible to VILI (Slutsky and Ranieri, 2000; Dreyfuss and Hubmayr, 2016). Various ventilation strategies have been proposed to manage ARDS and prevent VILI, but once underway it is difficult to break out of the vicious cycle of progressing injury (Slutsky and Ranieri, 2013).

We have shown in a number of studies in mice (Seah et al., 2011; Smith et al., 2013, 2017) that the nature and degree of lung injury is reflected in what we term the *derecruitability* of the lung. Derecruitability is measured by applying a recruitment maneuver (deep inflation) to maximally open closed airspaces followed immediately by a period (typically 3 min) of mechanical ventilation at a designated tidal volume (*V*_t_) and level of positive end-expiratory pressure (PEEP). Lung stiffness (*H*) rises progressively during the ventilation period to a degree that is modest (perhaps 20%) in a normal lung, but is dramatically enhanced in the injured lung to an extent that reflects the degree of injury (Smith et al., 2013). Some of the post-recruitment increase in *H* in the normal lung likely reflects changes in surface tension arising from the dynamics of surfactant at the air-liquid interface and viscoelastic adaptation of tissue stress, but the highly exaggerated increases in *H* seen in the injured lung are largely the result of progressive derecruitment of airspaces (Massa et al., 2008; Smith et al., 2017; Hamlington et al., 2018b).

We denote as *D*_*rate*_ the mean rate of increase in *H* over the 3 min following recruitment, while *H*_*1*_ is the first measurement of *H* made immediately after recruitment (Smith and Bates, 2013; Smith et al., 2013). Both *D*_*rate*_ and *H*_*1*_ increase with the severity of lung injury, but they do not mirror each other exactly. *D*_*rate*_ quantifies derecruitment dynamics that manifest over a timescale of minutes, probably reflecting instabilities in the fluid layer lining the small airways and alveoli (Smith et al., 2013). *H*_*1*_, on the other hand, quantifies either much more rapid derecruitment phenomena or an inability to recruit at all, more likely explicable on the basis of rapid alveolar flooding by accumulated edema fluid and/or unstable collapse of lung units caused by high surface tension in the air-liquid interface (Smith et al., 2013). *D*_*rate*_ and *H*_*1*_ together thus comprise a pair of sensitive biomarkers of lung injury (Smith et al., 2013).

We have previously shown in mice that, as VILI develops, the time-course of *H*_*1*_ can be accurately accounted for in terms of the accumulated leak of plasma-derived material through holes in the blood-gas barrier (Mori et al., 2018). These holes appear to be generated by both atelectrauma and volutrauma acting synergistically in a rich-get-richer fashion (Hamlington et al., 2018a). The rate of accumulation of edema in the airspaces, and thus the displacement of air that results, is a function of the number and sizes of these holes, both of which increase with time during injurious mechanical ventilation. In the present study, we extend this analysis to *D*_*rate*_ in order to establish a consistent theory of derecruitability of the injured lung that links both *H*_1_ and *D*_*rate*_ to the biophysical processes taking place during the development of VILI.

## Materials and Methods

### Model Development

In the following model development we take a similar analytical approach to our previous study of the dependence of *H*_*1*_ on degree of lung injury, this time focusing on the complementary parameter *D*_*rate*_. To account for the progressive increase in *D*_*rate*_ that accompanies the development of VILI, it is necessary to model events taking place over two different scales of time. The longer time scale, on the order to minutes to hours, concerns the progression of VILI itself. The shorter time scale, on the order of seconds to minutes, involves the dynamic recruitment and derecruitment of alveoli and small airways that are reflected in our periodic assessments of VILI. In order to avoid ambiguity between these two time scales in the following mathematical development, events taking place over the shorter time scale (seconds to a few minutes) will be referenced to time denoted by τ while events taking place over the longer time scale (many minutes to hours) will be referenced to time denoted by *t*.

We first consider the short time-scale assessment of VILI, which is based on the computational model of recruitment/derecruitment dynamics proposed by Bates and Irvin (2002) consisting of a parallel distribution of lung units. Each unit has its own airway to which is applied the same pressure. The units open and close according to their randomly assigned critical opening and closing airway pressures (*P*_*o*_ and *P*_*c*_, respectively). These pressures are drawn from probability distribution *f*_*P*__*o*_(*P*_*o*_) and *f*_*P*__*c*_(*P*_*c*_), respectively. However, a unit does not necessarily open or close as soon as it traverses its particular value of either *P*_*o*_ or *P*_*c*_. Rather, a latency is built into the process by associating with each unit a *virtual trajectory* variable *x* that can assume any value between 0 (corresponding to the unit being definitively closed) and 1 (definitively open). An open unit remains open until *x=0*, while a closed unit remains closed until *x=1*. Movement of *x* along the virtual trajectory occurs to the right (i.e., toward increasing values of *x*) when *P* > *P*_*o*_, and to the left when *P* < *P*_*c*_, with the rate of change *x* being proportional to the amount by which *P* either exceeds *P*_*o*_ or is less than *P*_*c*_. The constants of proportionality that determine the velocity of *x* rightward and leftward are *s*_*0*_ and *s*_*c*_, respectively. That is,

$$\frac{d\,x}{d\,\tau }\,\_=\left(P-P_{o}\right)\,s_{o};\text{for}\,P\geq P_{o}$$

$$=\left(P-P_{c}\right)\,s_{c};\text{for}\,P\leq P_{c}$$

(1) $$=0;\text{for}\,P_{c}<P<P_{o}$$

where 0≤*x* <  1. The velocity constants *s*_*0*_ and *s*_*c*_ for each unit are drawn from probability distributions *f*_*s*_*o*__(*s*_0_) and *f*_*s*_*c*__(*s*_*c*_), respectively.

Following the original model (Bates and Irvin, 2002), we assume that *P*_*o*_ and *P*_*c*_ are distributed according to Gaussians with variances σ_*0*_ and σ_*c*_, and means μ_*0*_ and μ_*c*_, respectively. In accord with the findings of Massa et al. (2008) in acid-injured mice, we set the two variances to be equal (i.e., σ_0_ = σ_*c*_ = σ) and the two means separated by δ*P* such that the opening pressures are greater than the closing pressures (i.e., μ_0_ = μ_*c*_ + δ*P*)

(2) $$f_{P_{c}}\,\left(P_{c}\right)=\frac{1}{\sigma \,\sqrt{2\,\pi }}\,e^{-\frac{1}{2}\,{\left(\frac{P_{c}-\mu_{c}}{\sigma }\right)}^{2}}$$

and

(3) $$f_{P_{o}}\,\left(P_{o}\right)=\frac{1}{\sigma \,\sqrt{2\,\pi }}\,e^{-\frac{1}{2}\,{\left(\frac{P_{o}-\mu_{c}-\delta \,P}{\sigma }\right)}^{2}}$$

We assume that σ and δ*P* do not change with increasing injury, so the progression of VILI is reflected entirely in the way that μ_*c*_ increases with time.

The velocity distributions *f*_*s*_*o*__(*s*_0_) and *f*_*s*_*c*__(*s*_*c*_) were originally specified on purely empirical grounds as conforming to hyperbolic functions (Bates and Irvin, 2002), motivated by the ubiquitous appearance of such functions in naturally occurring complex systems (West, 1990; Robertson et al., 2000; West et al., 2000). Solving the model analytically using such functions is not possible, however, because hyperbolas are not finite-integrable over *s* ∈ (0,∞), but finite integrals can be obtained by having the exponent of the power law be different from unity and by specifying a small positive lower limit, *s*_*m*_, for integration. Accordingly, we set

(4) $$f_{s_{c}}\,\left(s_{c}\right)=\frac{\alpha -1}{s_{m}}\,{\left(\frac{s_{c}}{s_{m}}\right)}^{-\alpha }$$

where 0 < *s*_*m*_ < *s*_*c*_ < ∞ and α > 1.

When modeling how the fraction of closed lung, *F*_*closed*_, changes during a derecruitability test, we avoid having to consider *f*_*s*_*o*__(*s*_0_) specifically by assuming, as did Albert et al. (2009), that it is only derecruitment that takes place to any significant extent during the 3 min following a recruitment maneuver. That is, we assume that the fully recruited lung derecruits progressively during these 3 min as if exposed to an effective pressure, *P*_*derecruit*_, that is fixed throughout the breath and that reflects the mean airway pressure applied by the mechanical ventilator. *P*_*derecruit*_ is determined by the applied level of PEEP plus some positive Δ*P* that depends on the ventilation regimen that is applied on top of the PEEP. As the lung becomes progressively more injured, and thus stiffer, mean airway pressure increases. This pressure increase is offset to some extent, however, by the compliance of the gas in the piston and connecting tubing of the flexiVent ventilator (Scireq, Montreal, Canada) that was used to collect the experimental data. While the effect of this compliance on calculation of *H* was compensated for digitally (Schuessler and Bates, 1995), it still absorbed an increasing fraction of the pressure increases as the lung stiffened (Smith et al., 2013). Mean airway pressure also increases somewhat over the course of a single derecruitability test. However, in order to obtain an analytical solution to our model we assumed that *P*_*derecruit*_ remains unchanged over each of the 3 min derecruitment maneuvers. This is clearly not precisely the case, but we take it to be a reasonable first-order approximation since tidal volume is quite low during the tests.

For a unit to have the possibility of closing during a derecruitability test it must therefore satisfy the condition *P*_*c*_ > *P*_derecruit_. Whether or not a particular unit actually closes during the test, however, depends on whether it moves along its virtual trajectory rapidly enough for *x* to reach a value of 0 before the 3 min test is complete and that there is no significant retrograde motion of *x* during the inspiratory portions of the test. If the duration of the test were to continue indefinitely, the asymptotic value to which *F*_*closed*_ would tend is that which satisfies the above condition, which is

(5) $$F_{\text{closed}}\,\left(\infty \right)=\int_{P_{\text{derecruit}}}^{\infty }\left[\frac{1}{\sigma \,\sqrt{2\,\pi }}\,e^{-\frac{1}{2}\,{\left(\frac{P_{c}-\mu_{c}}{\sigma }\right)}^{2}}\right]\,d\,P_{c}$$

This situation is never actually achieved, of course, because the derecruitability test is terminated after 3 min.

We assume that the recruitment maneuver establishes an initial condition for which all units open and are positioned at *x=1* along their respective virtual trajectories. As derecruitment proceeds during the test, the various units in the model close sequentially. The time, τ, at which a given unit closes is determined by how quickly it traverses the length of its virtual trajectory from *x=1* to *x=0*, which is given by

(6) $$\tau =\frac{1}{s_{c}\,\left|P_{\text{derecruit}}-P_{c}\right|}$$

which means that the units that have closed by time τ are those that have closing pressures satisfying $\frac{1}{\tau \,\left(P_{c}-P\right)}<s_{c}$. Assuming, as did Albert et al. (2009), that *f*_*P*_*c*__(*P*_*c*_) and *f*_*s*_*c*__(*s*_*c*_) are statistically independent, *F*_closed_(*P*,τ) is given by

(7) $$\begin{array}{cc} F_{\text{closed}}\,\left(P,\tau \right)=\int_{P}^{\infty }\int_{{\left[\left(P_{c}-P\right)\,\tau \right]}^{-1}}^{\infty }\frac{1}{\sigma \,\sqrt{2\,\pi }}\,e^{-\frac{1}{2}\,{\left(\frac{P_{c}-\mu_{c}}{\sigma }\right)}^{2}} & \\ \cdot \frac{\alpha -1}{s_{m}}\,{\left(\frac{s_{c}}{s_{m}}\right)}^{-\alpha }\,d\,P_{c}\,d\,s_{c} & \end{array}$$

A detailed step-by-step solution of Eq.7 is presented in the Supplementary Material.

Solving Eq. 7 we find that α = 2 and Eq. 4 of thus becomes

(8) $$f_{s_{c}}\,\left(s_{c}\right)=\frac{1}{s_{m}}\,{\left(\frac{s_{c}}{s_{m}}\right)}^{-2}$$

Massa et al. (2008) found σ = 3 in mice with acute lung injury caused by hydrochloric acid instillation. Smith et al. (2013) found μ_*c*_(*t*) = *kt*, where *k* is a constant, in injuriously ventilated mice. Since PEEP is zero and *H* is measured using small-amplitude perturbations in lung volume, we assume that *P=0* to a first approximation (Mori et al., 2018). *D*_*rate*_ can thus be written as:

(9) $$D_{\text{rate}}\,\left(t\right)=\frac{H_{1}\,\left(t\right)\,s_{m}}{\sqrt{2\,\pi }}\,\left[\sqrt{\frac{\pi }{2}}\,3\,k\,t\,\left(e\,r\,f\,\left(\frac{k\,t}{3\,\sqrt{2}}\right)+1\right)+9\,e^{-\frac{1}{2}\,{\left(\frac{k\,t}{3}\right)}^{2}}\right]$$

From our previous proposed model (Mori et al., 2018) we can relate Eq. 9 to *H*_*1*_ when *t* is sufficiently large:

(10) $$\lim_{t\to \infty }D_{\text{rate}}\,\left(t\right)=3\,k\,s_{m}\,\frac{1}{\sqrt{a}}\,\left(H_{1}\,\left(t\right)-\frac{H_{0}}{2}\right)$$

### Experimental Data

We tested the predictions of the above model against a set of data collected in mice and published previously (Smith et al., 2013). A detailed description of the experimental method used, as well as animals ethics approval, are given in this previous publication. Briefly, healthy 8- to 10-week-old female BALB/c mice (18.3–24.4 g) were anesthetized with 90 mg/kg intraperitoneal sodium pentobarbital and then connected to a computer-controlled mechanical ventilator (flexiVent, Scireq, Montreal, Canada). Following paralysis (0.5 ml/kg IP injection of pancuronium bromide) animals received a 21 min protocol starting with 16.5 min of injurious ventilation delivered at zero PEEP with a very large *V*_*t*_ (1.0, 1.1, 1.2, and 1.3 ml in 4 different groups of animals) followed by a derecruitability test (4.5 min protocol consisting of a deep inflation followed by ventilation at zero PEEP with *V*_*t*_ = 0.25 ml during which elastance was measured every 20 s). This 21 min protocol was repeated continuously for 4 h or until the animal died. The way that the lungs derecruit over time during the derecruitment test is reflected in the way that *H* increases with time (Allen and Bates, 2004). Finally, the resulting estimations of *D*_*rate*_ from the derecruitability tests were fitted to Eq. 15, and the 95% joint confidence regions for *s*_*m*_ and *k* were determined as described by Mori et al. (2018).

It should be pointed out that we also measured parameters related to the resistance of both the conducting airways and the respiratory system tissues in these mice. We focus here exclusively on the elastance parameter *H* in our model because it scales inversely with the fraction of lung that remains open, provided intrinsic tissue stiffness does not change, thus serving as a convenient biomarker of derecruitment. Lung tissue resistance scales similarly to elastance (Smith et al., 2020) and therefore adds no new information, so we did not include it in our model. Airway resistance tends to correlate less well with lung derecruitment because the proximal branches of the airway tree, which contribute much to airway resistance, mostly remain patent even when significant portions of the lung periphery derecruit (Smith et al., 2020).

### Model Fitting

The model described by Eq. 9 was fit to the elastance data versus time collected within each derecruitment maneuver with MATLAB (The Mathworks, Natick, MA, United States) using a non-linear least squares method (trust-region algorithm) for each animal individually in all 4 groups, resulting in a pair of values of *s*_*m*_ and *k* for each animal. The maximum number of evaluations and iterations were set to 600 and 400, respectively, and the termination tolerance value of the cost function (mean squared residual) was 10^–6^. Finally, the 95% joint confidence regions for *s*_*m*_ and *k* were determined as described by Mori et al. (2018).

## Results

**FIGURE 1:**
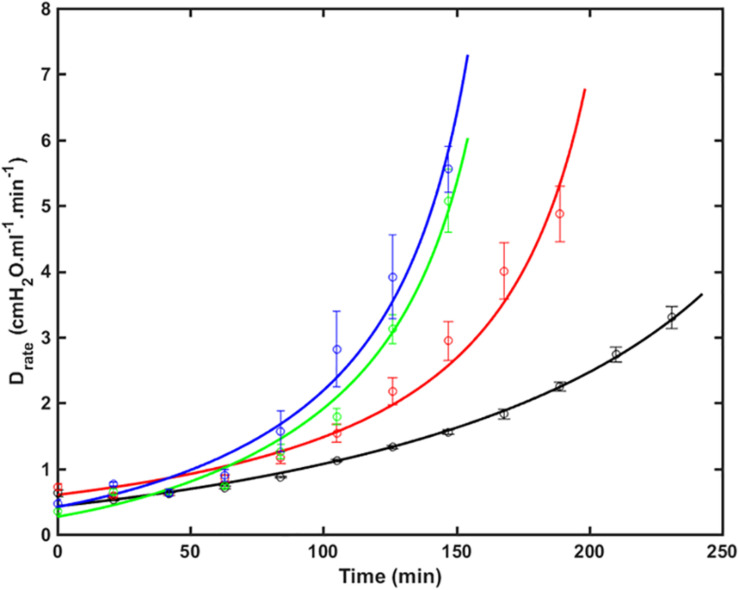
*D*_rate_ (average ± SE) versus time as VILI developed in the 4 groups of mice (symbols) together with the model fits to Eq. 9 (lines). Black: *V_t* = 1.0 ml (RMSE = 0.35); Red: *V_t* = 1.1 ml (RMSE = 0.64); Green: *V_t* = 1.2 ml (RMSE = 0.36); Blue: *V_t* = 1.3 ml (RMSE = 0.65). The data were collected in a previous study (Smith et al., 2013) (RMSE – root mean squared residual.).

Figure 1 shows the fits of Eq. 9 to experimental data from four groups of over-ventilated mice (*V*_*t*_ = 1.0, 1.1, 1.2, and 1.3 ml, with zero PEEP), together with the experimental data. The model fits pass within one standard error either side of the data points in most cases, and are very close to these ranges in the other cases. The *R*^2^ values for all fits were greater than 0.85. This demonstrates that the model accurately accounts for the way that injury, as reflected by *H*, accelerates over time and how this acceleration increases with increasing *V*_t_.

**FIGURE 2:**
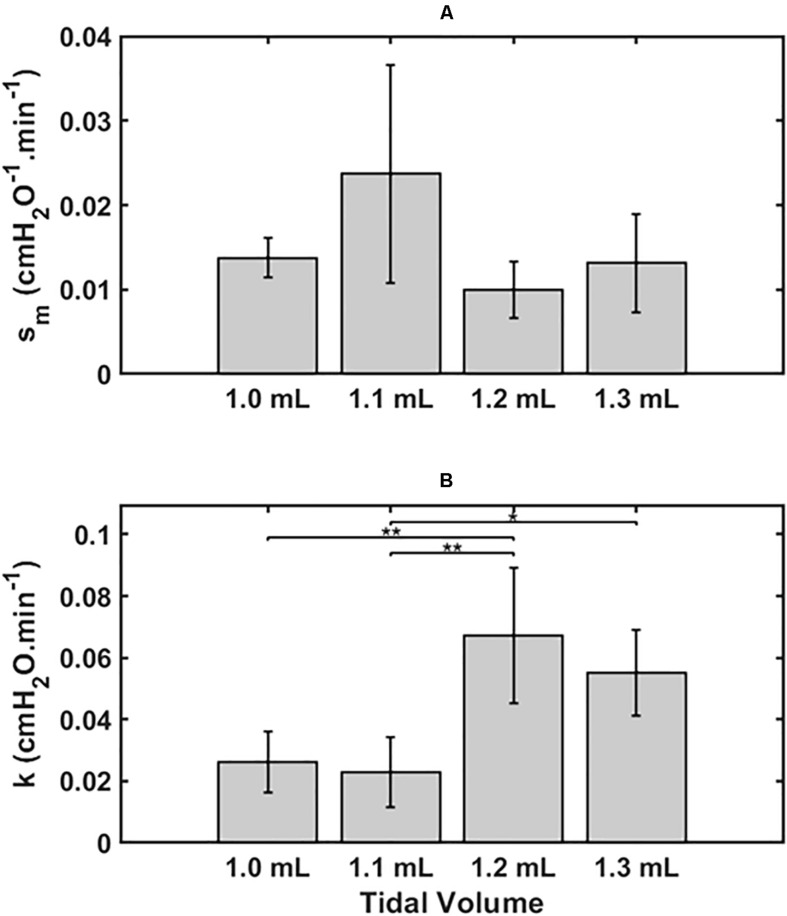
Mean ± 2 SEM for the two key model parameters *s_m* **(A)** and *k* **(B)** and for the 4 groups, 1.0 ml (*n* = 6), 1.1 ml (*n* = 6), 1.2 ml (*n* = 5) and 1.3 ml (*n* = 4). Statistically significant differences between groups are indicated by: *P < 0.05), **P < 0.01.

Figure 2 shows the two model parameters in Eq. 9, *s*_*m*_ and *k*, versus *V*_*t*_. There was no dependence of *s*_*m*_ on *V*_*t*_ by ANOVA (*p* = 0.11, Figure 2A), whereas *k* was significantly greater for the lungs exposed to the two higher *V*_*t*_ compared to the two lower *V*_*t*_ (*p* < 0.001, Figure 2B). Normality of the distributions of all parameter groups was supported by the Shapiro-Wilk test (*p* > 0.05). The Bonferroni *post hoc* test was performed to assess inter-groups differences for *k*. All statistical tests were performed in MATLAB 2019b (The Mathworks, Natick, MA, United States).

**FIGURE 3:**
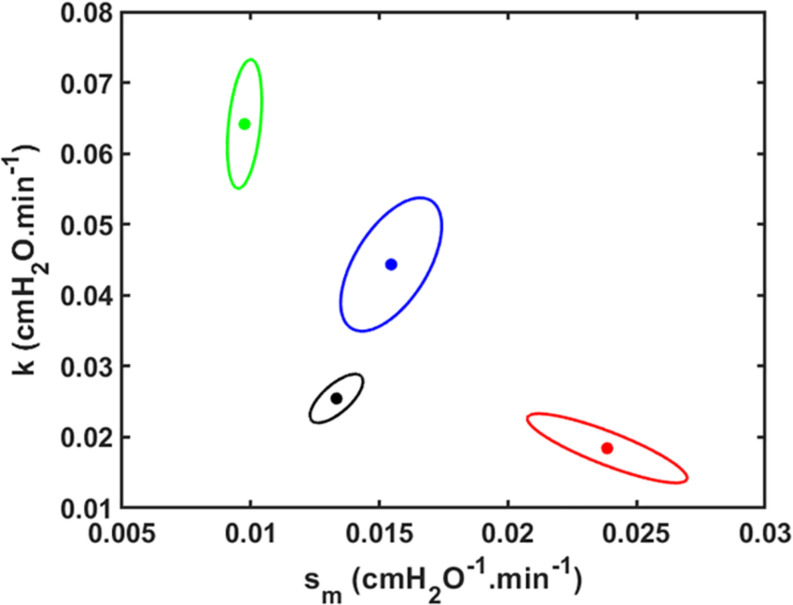
Joint paired 95% confidence regions for the model parameters. The optimum parameter values (those that minimize the root mean squared difference between measured and modeled *D*_rate_) are located at the centers of the ellipses. Black: *V_t* = 1.0 ml; Red: *V_t* = 1.1 ml; Green: *V_t* = 1.2 ml; Blue: *V_t* = 1.3 ml.

The joint 95% confidence intervals show clean separation between the parameter pairs for the four groups of mice (Figure 3).

**FIGURE 4:**
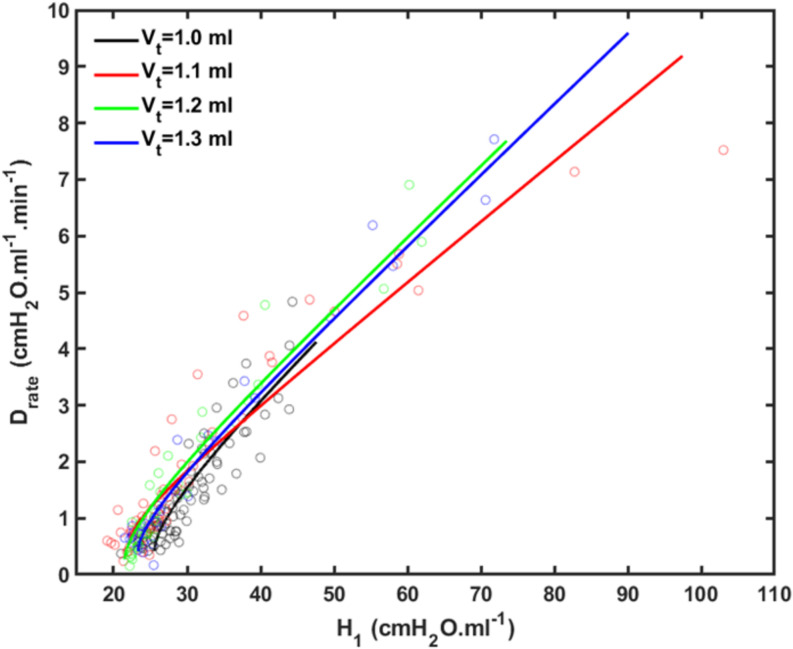
Rate of change of lung stiffness (*D*_rate_) versus initial measurement of post-recruitment stiffness (*H_1*) measured in the 4 groups of mice (symbols) together with the model fits (lines). Black: *V_t* = 1.0 ml; Red: *V_t* = 1.1 ml; Green: *V_t* = 1.2 ml; Blue: *V_t* = 1.3 ml.

**FIGURE 5:**
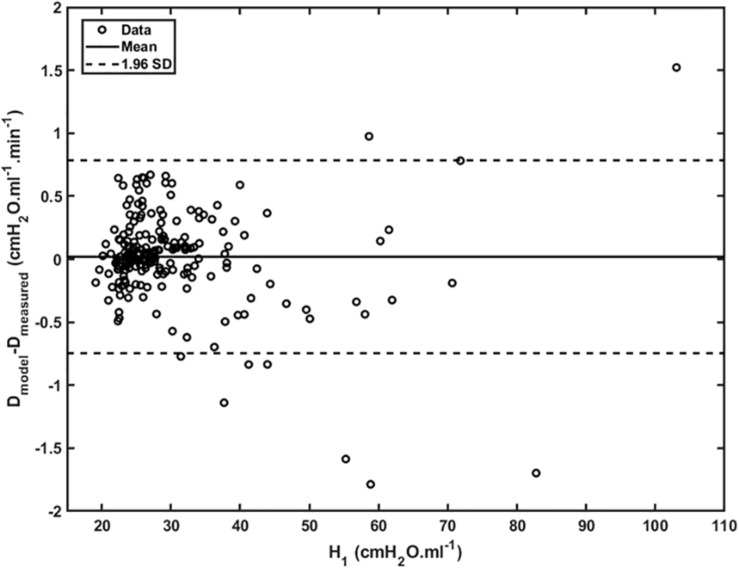
Difference between modeled and measured *D*_rate_ as a function of *H_1* (open dots). Mean is represented by the full line whereas 95% interval (± 1.96 SD) is represented by dashed lines.

Figure 4 shows plots of *D*_rate_(*t*) versus *H*_*1*_ for all animals studied. Also shown are the model predictions of *D*_rate_(*t*) from Eq. 9 versus *H*_*1*_ calculated according to Mori et al. (2018). Lastly, Figure 5 shows, in a Bland-Altman plot, the difference between modeled and measured *D*_*rate*_ values as a function of *H*_1_.

## Discussion

We have developed an analytical model of the time-course of derecruitment in the injured lung that mimics observations of how lung derecruitability evolves during the development of VILI. This analytical model is based on our previous numerical approach of ascribing recruitment and derecruitment dynamics to the behavior of a virtual trajectory associated with various lung units (Bates and Irvin, 2002; Massa et al., 2008). This model uses a similar analytic approach to our recent study that examined how *H* measured immediately after a recruitment maneuver (*H*_*1*_) increases as a function of worsening lung injury (Mori et al., 2018). In this sense, the present study complements our previous model analysis since together they provide a complete analytic description of how a deep inflation of the injured lung recruits closed lung units and then how these units derecruit again over time as governed by the values of the three fixed parameters α, *s*_*m*_, and *k*, where α and *k* depend on *V*_*t*_. The model of the present study accurately predicts the manner in which the temporal evolution of *D*_*rate*_ varies with both time and injurious *V*_*t*_ (Figure 1), dependencies that are embodied in a single parameter, *k*, thought to largely reflect how surface tension increases with time (Figure 2). Surface tension elevation is a consequence of surfactant dysfunction, itself caused either by direct damage to the surfactant or by the presence of material that has leaked from the vasculature into the airspaces of the lung. *H* can also increase as a result of accumulated fluid that floods alveoli. This model thus links developments in physical injury of the parenchyma tissue to the dynamics of lung function derangement via the physiologic mechanism of airspace derecruitment.

Analytical models generally do not have the same flexibility as numerical models to describe the complex details of experimental data. On the other hand, when the model equations are based on putative underlying mechanisms, as in the present case, analytical models may offer insight of a general nature that more empirical nature of numerical models cannot. In this regard, the model in the present study supports the suitability of a power-law function as describing the velocity distribution along virtual trajectories (Eq. 4). The parameter *s*_*m*_ sets the minimum closing velocity, which corresponds to those units that close so slowly they remain open throughout the entire derecruitability test. It is possible that these units would eventually close if the derecruitability tested lasted long enough, but they make no contribution to the data when the tests last only 3 min. Interestingly, the exponent of the power law was found to be −2 purely on the basis of the empirically observed linear increase in lung stiffness following a recruitment maneuver at zero PEEP. In other words, this exponent value comes from the qualitative behavior of the data rather than the fit of a particular model. This is significant because power laws have been invoked in a previous model of lung recruitment during lung inflation to account for avalanches of sequential opening events along the airway tree that are triggered when critical opening pressures are surpassed (Suki et al., 1994, 2000). Moreover, both inter-crackle intervals and terminal air space sizes that are linked by avalanches throughout the airway tree are characterized by power-law distributions having an exponent of −2 (Suki et al., 2000; Alencar et al., 2001). The similarity between the probability distribution for *s*_*c*_ and these other avalanche-based mechanisms in the lung might not be a coincidence, but rather might follow in some way from the fractal branching structure of the airway tree. Thus, although our model consists of a parallel collection of respiratory units that cannot open in the kinds of cascades observed in the airway tree (Suki, 2002), the fractal structure of the lung is embedded in our model through the power-law form of the distribution of *s*_*c*_ (Eq. 4) and thus conforms to previous work by Suki et al. (1994, 2000), Alencar et al. (2001), and Suki (2002).

Having the geometry of the airway tree be implicit in our model in the functional form for *s*_*c*_ could also explain the findings of Massa et al. (2008) that *f*_*s*_*o*__(*s*_0_) and *f*_*s*_*c*__(*s*_*c*_) are unchanged following lung injury. That is, although lung injury would be expected to damage the alveolar parenchyma and the airway epithelium, there is no reason to suppose it would alter the branching structure of the airway tree, which would leave the functional form for *s*_*c*_ preserved. On the other hand, we do expect that injury would increase the mean values of the distributions for *P*_*c*_ and *P*_*o*_ (Eqs 2 and 3, respectively) on the basis of increases in surface tension at the air-liquid interface within the lungs as a result of blood-gas barrier perforation and the subsequent airspace accumulation of fluid and protein (Massa et al., 2008; Smith et al., 2013). This conveniently allows us to account for the progression of lung injury largely in terms of the evolution of the value of a single parameter, *k* in Eq. 9 (Figures 2, 3).

We previously reported an apparent master relationship between and *D*_*rate*_ and *H*_*1*_ (Smith et al., 2013). The model developed in the present study, however, suggests this is not quite the case because rate of change of *D*_*rate*_ with injury (Eq. 10) is predicted to depend on *k*, which depends on *V*_*t*_ (Figure 2B), even though the initial value of *D*_*rate*_ (Eq. 10 of the Supplementary Material) does not (Figure 2A). On the other hand, the dependence of *dD*_rate_/*dH*_0_ on *V*_*t*_ is rather subtle (Figure 4), and is easily masked by noise in the data, so the notion of a single master relationship may still be a useful approximation. Also, both *H*_*1*_ (Mori et al., 2018) and *D*_*rate*_ (Figure 4) exhibit exponential growth over the time-scale of our experiment (up to 4 h), but they are unlikely to continue to increase indefinitely beyond this point because this would correspond to derecruitment of the entire lung. Even if this was a possibility, however, forcing a sizeable *V*_*t*_ into an ever shrinking lung would eventually lead to pressures that would cause rupture of the tissues. In any case, it appears that neither eventually would ever be realized because the mice died when about 75% of the lung became derecruited, at which point *H*_*1*_ and *D*_*rate*_ were still increasing exponentially.

Another feature of the relationship between *D*_*rate*_ and *H*_*1*_ is the early transient seen in Figure 4, which shows that *D*_*rate*_ to begin to increase early in the development of lung injury before *H*_*1*_ changes noticeably. We have previously postulated (Smith et al., 2013) that increases in *D*_*rate*_ are due to increases in surface tension that give rise to derecruitment dynamics manifesting over a timescale of minutes, while increases in *H*_*1*_ reflect flooded alveoli that have become permanently derecruited. This being the case, the relationship between *D*_*rate*_ and *H*_*1*_ in Figure 4 suggests that surfactant deactivation by plasma-derived fluid and proteins starts to occur as soon as this material arrives in the airspaces whereas a certain volume of fluid as to accumulate in the airspaces before it can cause noticeable alveolar flooding. It also suggests that *D*_*rate*_ is more directly related to the “rich-get-richer” hypothesis of VILI that we have previously advanced (Hamlington et al., 2018a; Mori et al., 2018) and thus may be the more sensitive marker of the onset of lung injury. On the other hand, even though the model errors for *D*_*rate*_ do not appear to depend on *H*_*1*_ (Figure 5), at large *t*, *D*_*rate*_, and *H*_*1*_ become linearly dependent on each other (Eq. 10) suggesting that both variables ultimately embody the same pathophysiological information, although the fits of the present study are not as good at this end of the data range compared to those of our previous study (Smith et al., 2013) which curve over and follow the data better. Regardless, both *D*_*rate*_ and *H*_*1*_ are increased by fluid accumulation in the airspaces, so some kind of monotonic relationship between the two parameters is to be expected.

Our model has a number of limitations. For example, although we have characterized derecruitability of the lung in terms of only two parameters, *H*_*1*_ and *D*_*rate*_, the increase in *H* with time during a derecruitability test is only approximately linear in its early stages; eventually, *H* begins to plateau (Allen et al., 2002; Allen and Bates, 2004; Massa et al., 2008). Such a plateau is inevitable as only those lung units for which *P* < *P*_*c*_ will eventually close as τ→∞. *D*_*rate*_ is thus an empirical characterization of only part of a more complex relationship. We also neglected intra-breath variations in the pressures applied to the lungs by mechanical ventilation, as well as the way they depend on the degree of lung injury. We assumed a constant pressure for derecruitment in order to be able to solve the model equations analytically (Eq. 7) and so gain the benefits of insight that a closed-form solution provides. In reality of course, these pressures are not constant because mean airway pressure rises for a given *V*_*t*_ as the lung becomes progressively more consolidated, so more quantitatively accurate predictions would require the numerical solution of a computational model in which airway pressures are allowed to vary in a realistic manner. This may partly explain why the ellipse areas of the joint parameter confidence regions in Figure 3 tend to be greater for larger *V*_*t*_ (i.e., greater VILI); we would generally expect a simple analytical model to fit better to data from normal or mildly injured lungs compared to those that are severely injured. We also waived details concerning alveolar micromechanics such as interactions between neighboring respiratory units, although we recognize the potentially important role of such interactions in lung injury (Knudsen and Ochs, 2018). Finally, we have assumed that the dynamics of derecruitability in the lung are accurate indicators of the degree of lung injury, but they are not direct measures of such. Nevertheless, numerous prior studies from our laboratory have established a tight link between derecruitability and direct measures of damage to the blood-gas barrier (Allen and Bates, 2004; Allen et al., 2005; Smith et al., 2017; Hamlington et al., 2018a,b).

In summary, we have developed an analytical version of our previously developed computational model of the dynamics of lung derecruitment following a recruitment maneuver. We found that the distribution of velocity constants defining rates of movement along the virtual trajectories associated with the pulmonary airways follows a power-law with the same exponent of −2 as found in other investigations into the dynamics of lung recruitment and derecruitment. We speculate that this may reflect the fractal-like structure of the airway tree throughout which recruitment and derecruitment may take place as cascades of open and closing events. Our study also suggests that the rate of change in lung stiffness represented by the parameter *D*_*rate*_, to the extent that this remains constant during a 3 min derecruitability test, is a particularly sensitive marker of the degree of lung injury.

## Data Availability Statement

The datasets generated for this study are available on request to the corresponding author.

## Ethics Statement

The animal study was reviewed and approved by Institutional Animal Care and Use Committee of the University of Vermont.

## Author Contributions

VM developed the model analysis, coded the simulations, and drafted the manuscript. BJS, BS, and JB contributed to the development of the model concept, checked the mathematics, and edited and approved the manuscript. All authors contributed to the article and approved the submitted version.

## Conflict of Interest

BJS and JB filed the following patent that is potentially related to the subject matter of the manuscript: “Variable ventilation as a diagnostic tool for assessing lung mechanical function” JB and BJS. PCT Application WO2015127377 A1, Filed on February 23, 2014 (C538). The remaining authors declare that the research was conducted in the absence of any commercial or financial relationships that could be construed as a potential conflict of interest.
