# Supplementary material for: Modeling Lung Derecruitment in VILI Due to Fluid-Occlusion: The Role of Emergent Behavior
Source: Front Physiol. 2020 Oct 30;11:542744. doi: 10.3389/fphys.2020.542744 (PMC7662071; doi:10.3389/fphys.2020.542744)
Supplement: Supplementary file 1 [file Table_1.DOCX]

**SUPPLEMENTAL MATERIAL**

**Modeling lung derecruitment in VILI due to fluid-occlusion: An analytical approach**

Vitor Mori^1,2*^, Bradford J. Smith^3^, Bela Suki^4^ and Jason H. T. Bates^1^

^1^Vermont Lung Center, Department of Medicine, University of Vermont College of Medicine, Burlington, VT 05405, USA

^2^Department of Telecommunications and Control Engineering, University of Sao Paulo, Sao Paulo, Brazil

^3^Department of Bioengineering, University of Colorado Denver | Anschutz Medical Campus, Aurora CO 80045, USA

^4^ Department of Biomedical Engineering, Boston University, Boston MA 02215, USA

Short title: **Derecruitment in VILI**

*Corresponding Author:

Vitor Mori

149 Beaumont Ave, HSRF Room 233

Burlington, VT 05405

Tel: (802) 355-2485

Email: vitor.mori@uvm.edu

**Detailed Model Derivation**

From Eq. 7 of the manuscript, we have:

| $F_{closed}\left( P,\tau\right)=\int_{P}^{\infty} \int_{\left[ \left( P_{c}-P \right)\tau\right]^{-1}}^{\infty} \frac{1}{\sigma\sqrt{2\pi}}e^{-{\frac{1}{2}\left( \frac{P_{c}-\mu_{c}}{\sigma} \right)}^{2}}\cdot\frac{\alpha-1}{s_{m}}\left( \frac{s_{c}}{s_{m}} \right)^{-\alpha}dP_{c}ds_{c}$ | (1) |
| --- | --- |

where we assume that $s_{m}$ is less than the lower limit of the inner integral. This gives

| $F_{closed}\left( P,\tau\right)=\frac{\beta(P){s_{m}}^{\alpha-1}}{\sigma\sqrt{2\pi}}\cdot\tau^{\alpha-1}$ | (2) |
| --- | --- |

with

| $\beta(P)=\int_{P}^{\infty} \left( P_{c}-P \right)^{\alpha-1}\cdot e^{-{\frac{1}{2}\left( \frac{P_{c}-\mu}{\sigma} \right)}^{2}}dP_{c}$ | (3) |
| --- | --- |

We assume that the way that lung elastance $H(t,\tau)$ changes with time $\tau$ following a recruitment maneuver performed at time $t$ is due entirely to derecruitment, which means that

| $H\left( t,\tau\right)=\frac{H_{1}\left( t \right)}{1-F_{closed}\left( P,\tau\right)}$ | (4) |
| --- | --- |

where $H_{1}\left( t \right)$ is elastance immediately following the derecruitment maneuver. Therefore, the rate of change of elastance following the recruitment maneuver is

| $D_{rate}\left( P,t,\tau\right)=\frac{\partial}{\partial\tau}\left[ \frac{H_{1}\left( t \right)}{1-F_{closed}\left( P,\tau\right)} \right]=\frac{H_{1}\left( t \right)}{\left( 1-F_{closed}\left( P,\tau\right) \right)^{2}}\cdot\frac{{s_{m}}^{\alpha-1}}{\sigma\sqrt{2\pi}}\cdot\beta(P)\left( \alpha-1 \right)\tau^{\alpha-2}$ | (5) |
| --- | --- |

Following a recruitment maneuver, $H\left( t,\tau\right)$ increases monotonically with $\tau$ toward an apparent asymptote and thus has a downward concavity, but intially its increase is approximately linear (Massa et al., 2008;Smith et al., 2013). For small $\tau$, therefore, $D_{rate}\left( P,t,\tau\right)$ is independent of $\tau$ and $\left( 1-F_{closed}\left( P,\tau\right) \right)^{2}\approx1$, in which case Eq. 5 reduces to

| $D_{rate}\left( P,t \right)=H_{1}\left( t \right)\cdot\frac{{s_{m}}^{\alpha-1}}{\sigma\sqrt{2\pi}}\cdot\beta(P)\left( \alpha-1 \right)\tau^{\alpha-2}$ | (6) |
| --- | --- |

which implies that $\alpha=2$. Equation Eq. 4 of the manuscript thus becomes

| $f_{s_{c}}(s_{c})=\frac{1}{s_{m}}\left( \frac{s_{c}}{s_{m}} \right)^{-2}$ | (7) |
| --- | --- |

and so

| $D_{rate}\left( P,t \right)=\frac{{H_{1}\left( t \right) s}_{m}}{\sqrt{2\pi}}\left[ \sqrt{\frac{\pi}{2}}\sigma\left( \mu_{c}-P \right)\left( erf\left( \frac{\mu_{c}-P}{\sqrt{2}\sigma} \right)+1 \right)+\sigma^{2}e^{-\frac{1}{2}\left( \frac{\mu_{c}-P}{\sigma} \right)^{2}} \right]$ | (8) |
| --- | --- |

Massa *et al.* (Massa et al., 2008) found $\sigma=3$ in mice with acute lung injury caused by hydrochloric acid instillation. Smith *et al.* (Smith et al., 2013) found $\mu_{c}\left( t \right)=kt$, where $k$ is a constant, in injuriously ventilated mice. Since PEEP is zero and $H$ is measured using small-amplitude perturbations in lung volume, we assume that $P=0$ to a first approximation (Mori et al., 2018). Equation 8 can thus be written

| $D_{rate}\left( t \right)=\frac{{H_{1}\left( t \right) s}_{m}}{\sqrt{2\pi}}\left[ \sqrt{\frac{\pi}{2}}3kt\left( erf\left( \frac{kt}{3\sqrt{2}} \right)+1 \right)+9e^{-\frac{1}{2}\left( \frac{kt}{3} \right)^{2}} \right]$ | (9) |
| --- | --- |

### Inspection of Eq. 9 shows that, at $\boldsymbol{t=0}$ when the lungs are normal,

| $\boldsymbol{D}_{\boldsymbol{rate}}\boldsymbol{(0)=}\frac{\boldsymbol{9}{\boldsymbol{H}_{\boldsymbol{1}}\left( \boldsymbol{0} \right)\boldsymbol{s}}_{\boldsymbol{m}}}{\sqrt{\boldsymbol{2}\boldsymbol{\pi}}}$ | (10) |
| --- | --- |

### As $\boldsymbol{t}$ increases, the last term in the square brackets in Eq. 9 decays toward zero and $\boldsymbol{D}_{\boldsymbol{rate}}\boldsymbol{(t)}$ asymptotes toward

| $\lim_{t\to\infty} D_{rate}\left( t \right)={3kts}_{\boldsymbol{m}}H_{1}\left( t \right)$ | (11) |
| --- | --- |

### We showed in our previous modeling study (Mori et al., 2018) that

| $H_{1}\left( t \right)=\frac{H_{0}}{1-at^{2}}$ | (12) |
| --- | --- |

where $H_{0}=H_{1}(0)$ and $a$ is a constant. Solving Eq. 12 for $t$ and substituting into Eq. 11 gives

| $t=\sqrt{\frac{1}{a}\left( 1-\frac{H_{0}}{H_{1}(t)} \right)}$ | (13) |
| --- | --- |

$H_{1}$ becomes substantially larger than $H_{0}$ when $t$ becomes large and substantial lung injury develops, in which case we can say

| $t\approx\frac{1}{\sqrt{a}}\left( 1-\frac{H_{0}}{{2H}_{1}(t)} \right)$ | (14) |
| --- | --- |

### Substituting this into Eq. 11 then gives

| $\lim_{t\to\infty} D_{rate}\left( t \right)=3ks_{\boldsymbol{m}}\frac{1}{\sqrt{a}}\left( H_{1}\left( t \right)-\frac{H_{0}}{2} \right)$ | (15) |
| --- | --- |
